# Evaluating pregnancy termination rates for fetal chromosome and single gene disorders

**DOI:** 10.1002/jgc4.70099

**Published:** 2025-08-15

**Authors:** Madeline J. Herman, Emily B. Rosenfeld, Nicole Kasatkin, Gary A. Heiman, Shama P. Khan, Elena Ashkinadze

**Affiliations:** ^1^ Ann B. Barshinger Cancer Institute Penn Medicine Lancaster General Health Lancaster Pennsylvania USA; ^2^ Rutgers University Genetic Counseling Master's Program Piscataway New Jersey USA; ^3^ Division of Maternal‐Fetal Medicine, Department of Obstetrics, Gynecology, and Reproductive Sciences Rutgers Robert Wood Johnson Medical School New Brunswick New Jersey USA; ^4^ Department of Genetics and the Human Genetics Institute of New Jersey Rutgers, the State University of New Jersey Piscataway New Jersey USA

**Keywords:** amniocentesis, chorionic villus sampling, chromosome disorders, fetal genetic analysis, genetic counseling, prenatal diagnosis, single gene disorders

## Abstract

We report pregnancy termination rates following a variety of fetal diagnoses and determine which factors may influence this decision. We conducted a retrospective chart review of pregnancies diagnosed with a genetic abnormality at a single institution from January 2012 to April 2023. The type of diagnosis, termination status, and thirteen demographic factors were collected. The primary outcome assessed was whether or not an individual terminated the pregnancy. Data analysis consisted of multivariable logistic regression. Of the 2120 patients who underwent prenatal diagnostic testing, 332 received a fetal diagnosis and met inclusion criteria. The overall termination rate was 61.5% (204/332). Compared with sex chromosome abnormalities, trisomy 18/trisomy 13/triploidy (adjusted odds ratio [aOR] 6.35, 95% Confidence Interval [CI] 1.93–20.90) and trisomy 21 (aOR 4.39, 95% CI 1.58–12.24) had higher odds to terminate, while likely benign copy number variants (CNVs) (aOR 0.17, 95% CI 0.03–0.99) had lower odds to terminate. Black paternal race and ethnicity had a lower termination rate (aOR 0.08, 95% CI 0.03–0.23) compared to White counterparts. Earlier gestational age at diagnosis was associated with higher odds of termination (aOR 0.84, 95% CI 0.78–0.90). This study demonstrates that termination rates varied by type of fetal diagnosis. Paternal race and ethnicity, as well as gestational age at diagnosis, also impacted the decision to terminate.

**What is already known about this topic?**

Termination rates have been examined in the context of common aneuploidies. Advancements in prenatal screening have led to increases in diagnostic testing and prenatal genetic diagnoses that have rarely been studied, such as copy number variants and single gene disorders.

**What does this study add?**

Termination rates of a variety of fetal diagnoses are reported, including aneuploidies, copy number variants, and single‐gene disorders. Factors are identified that impact the decision to terminate an affected pregnancy.

## 1 INTRODUCTION

The ability to detect fetal chromosome anomalies has been available since the advent of amniocentesis and its acceptance as a standard of care in the 1970s (Carlson & Vora, 2017). The introduction of chorionic villus sampling (CVS) in 1983 provided the opportunity for earlier fetal diagnosis (Ward et al., 1983). Standard testing following a prenatal diagnostic procedure began with karyotype analysis. Since 2016, the addition of prenatal microarray testing has routinely become available and identifies a clinically significant copy number variant (CNV) in 1.7% of average‐risk pregnancies (American College of Obstetricians and Gynecologists, 2016; Wapner et al., 2012). Additionally, sequencing technologies allow for the diagnosis of single‐gene disorders identified on expanded carrier screening (American College of Obstetricians and Gynecologists, 2016). These novel technologies allow for the prediction of a broader number of abnormalities affecting a pregnancy, including aneuploidy, CNVs, and single‐gene disorders.

Not only has the ability to prenatally diagnose genetic abnormalities grown, but so has the ability to alert those at higher risk through screening. Population screening for aneuploidy has expanded from maternal serum assays to prenatal cell‐free DNA (cfDNA) screening, allowing for broader detection of pregnancies at an increased risk for chromosomal abnormalities (American College of Obstetricians and Gynecologists, 2020). The use of expanded carrier screening has allowed couples to know their risk for single‐gene disorders before a diagnosis in the family (American College of Obstetricians and Gynecologists, 2017).

After receiving a fetal diagnosis, some individuals choose to terminate the affected pregnancy. In the United States of America, termination of pregnancy became a protected right for individuals under Roe v. Wade (1973). This decision stated that termination of pregnancy during the first trimester was allowed in all states with no state‐instituted regulations. States were allowed to institute their own regulations and laws regarding pregnancy termination during the second and third trimesters. In 2022, Roe v. Wade (1973) was overturned through Dobbs v. Jackson Women's Health Organization (2022) and termination of pregnancy was no longer a protected right nationwide. Regulations and laws regarding termination at all stages of pregnancy became entirely state determined.

The State Constitution of New Jersey protected freedom of reproductive rights, including termination of pregnancy during the protections of Roe v Wade and following its overturn. The Freedom of Reproductive Choice Act, P.L. (2021) maintains those reproductive protections in the state of New Jersey. The option to terminate a pregnancy is available in New Jersey at any gestational age for any reason that an individual is seeking a termination. However, some institutions in New Jersey limit their availability to the first and second trimester of pregnancy.

For many years, prior research has examined termination rates in the context of common aneuploidies. Severity of the fetal diagnosis, history of a previous termination, type of prenatal diagnostic procedure, and ethnicity have been shown to significantly impact the decision to terminate a pregnancy affected with a common aneuploidy (Hawkins et al., 2013; Hume & Chasen, 2015; Shaffer et al., 2006; Suzumori et al., 2015). Only recently have studies begun to assess termination rates in the context of other indications, such as CNVs and single‐gene disorders (Bruwer et al., 2022; Shi et al., 2023; Stern et al., 2021). Similarly, severity of diagnosis for CNVs and single‐gene disorders has also been reported as a significant predictor of the decision to terminate in other studies outside of common aneuploidies (Shi et al., 2023; Stern et al., 2021), with other predicting factors just beginning to be reported including the presence of ultrasound anomalies (Shi et al., 2023).

As the types of fetal diagnoses that are identified expand due to advancements in prenatal screening and diagnostic testing, the factors influencing the decision to terminate an affected pregnancy need to be re‐examined. Having a better understanding of the factors that influence this decision can be impactful for healthcare providers and ultimately allow for improvements in patient care. The objective of this study was to investigate pregnancy termination rates for fetal chromosome and single gene disorders after fetal diagnosis and their correlation with clinical and demographic variables.

## 2 METHODS

We conducted a retrospective chart review from January 1, 2012, through April 1, 2023. This timeframe was selected due to limited availability of chart reviews prior to 2012 and the data collection period for the researchers closing in April 2023. Those considered for this study had an amniocentesis or CVS, received an abnormal genetic result, and were seen for genetic counseling at a tertiary referral center in New Jersey. Subjects were included in this study if they had a singleton intrauterine viable pregnancy at the time of result disclosure. Subjects were excluded if they had a multifetal pregnancy, terminated before result disclosure, or had a nonviable pregnancy at the time of result disclosure.

Following an Institutional Review Board approval of the protocol, subjects who met inclusion criteria were identified through an internal database that included all amniocentesis and CVS procedures performed at this facility. Chart reviews were conducted using electronic medical record systems, Epic and Centricity. Information extracted included the type of fetal diagnosis, whether the patient chose to continue or terminate the pregnancy, and potential covariate information. Biases were minimized through uniform data abstraction and multivariable logistic regression to account for other variables. The Strengthening the Reporting of Observational Studies in Epidemiology (STROBE) reporting guidelines were followed (von Elm et al., 2007).

The type of diagnosis was considered an independent variable that was a potential predictor of the outcome. The fetal diagnoses were recorded (Table A1) and categorized as follows: trisomy 21, trisomy 18/trisomy 13/triploidy, sex chromosome abnormalities, balanced rearrangements, unbalanced rearrangements/rare trisomies, profound/severe single‐gene disorders, moderate/mild single‐gene disorders, copy number variants (CNVs) with a known clinical phenotype, CNVs with a variable phenotype, CNVs with a likely benign phenotype, regions of homozygosity, and cytomegalovirus. These categories were selected based on similarity in phenotype implications; for example, unbalanced rearrangements were grouped with rare trisomies due to their similarity in symptom severity and life expectancy. The single‐gene disorders were categorized into profound/severe or moderate/mild following the disease severity classifications proposed by Lazarin et al. (2014).

The outcome was a binary variable defined as whether the patient elected to continue or terminate the pregnancy. Another thirteen variables were analyzed as potential covariates (Table 1). The categorization of race and ethnicity follows guidelines proposed by the National Institutes of Health (United States Department of Health and Human Services, National Institutes of Health, 2015).

**TABLE 1:** Potential covariates analyzed.

Covariate	Categories
History of a previous termination	Yes or no
History of two or more miscarriages	Yes or no
Family history of genetic disease in a first, second, or third‐degree relative	Yes or no
Whether there was a results disclosure with a genetic counselor	Yes or no
Type of diagnostic procedure	Chorionic villus sampling (CVS) or amniocentesis
Gestational age at diagnosis	Number of weeks
Ultrasound anomalies	Soft marker(s), single anomaly, multiple anomalies, or none
Maternal race and ethnicity^a^	Asian, Asian American, or Pacific Islanders [AAPI], Black, Hispanic, Middle East or North African [MENA], White, or unknown
Paternal race and ethnicity^a^	Asian, Asian American, or Pacific Islanders [AAPI], Black, Hispanic, Middle East or North African [MENA], White, or unknown
Maternal age	<35, ≥35, or unknown
Paternal age	<40, ≥40, or unknown
Number of living children	Multiple, one, or none
Indication for diagnostic procedure	Abnormal antenatal screen, positive carrier screening, abnormal ultrasound findings, or information seekers

^a^ The categorization of race and ethnicity followed the single self‐reported race and ethnicity documented in the chart.

Descriptive statistics (frequencies of categorical responses and means of continuous variables) were calculated. Using multivariable logistic regression, we developed a model to determine if the type of fetal diagnosis (independent variable) was associated with elective termination (dependent variable), controlling for potential confounders. In this model, each type of fetal diagnosis was compared to sex chromosome abnormalities. Each potential confounder was individually tested against the independent variable and dependent variable. The potential confounders were then added to this model to assess if the relationship between the independent and dependent variables was altered. Backward elimination was performed to determine a parsimonious final model, using the Akaike Information Criterion (AIC) as the stopping rule (Dziak et al., 2020). The ability of the final model to discriminate between those who chose to terminate and those who did not was calculated by the area under the receiver‐operating characteristic (AUC), where an AUC value of 0.5 represents a model with no discriminative ability and an AUC of 1.0 represents a model with perfect discriminative ability (Steyerberg et al., 2010). All data analysis was conducted using Stata statistical software (StataCorp LLC, 2021).

## 3 RESULTS

**FIGURE 1:**
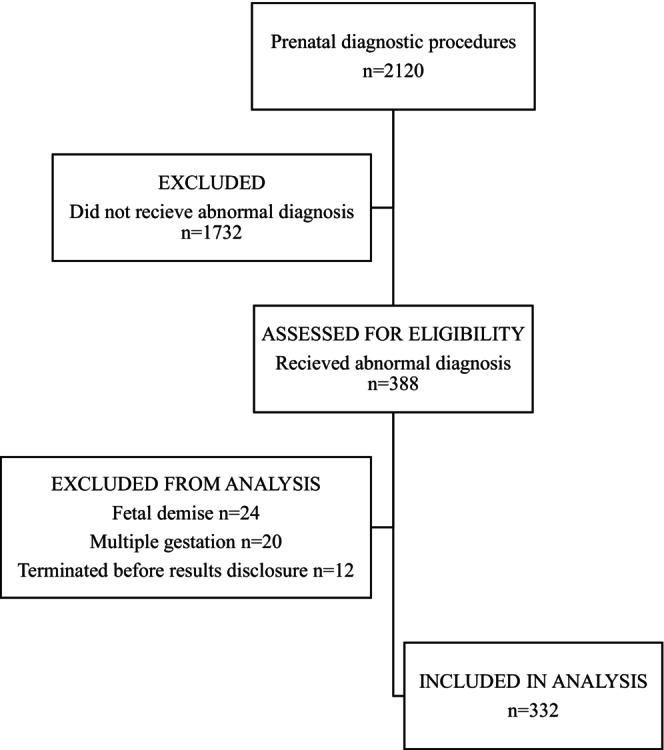
Flowsheet demonstrating patient inclusion and exclusion criteria.

During the study period, 2120 patients underwent a diagnostic prenatal procedure. A total of 388 of those patients received a fetal diagnosis. Of those, 56 patients were excluded from the analysis due to a multiple gestation pregnancy (*n* = 20), fetal demise before result disclosure (*n* = 24), and termination before result disclosure (*n* = 12). The remaining 332 patients met the inclusion criteria and were incorporated into the analysis (Figure 1).

Nearly two‐thirds (*n* = 204, 61.5%) pursued termination of pregnancy for a fetal diagnosis. Termination rates among each of the potential covariates in this study were collected (Table 2). Of those that terminated, the majority were of advanced maternal age (≥35 years) (56.4%), were of paternal age <40 years (71.6%), had a diagnosis via CVS (70.1%), had no history of previous terminations (77.9%), had no history of >2 spontaneous abortions (87.8%), and had no family history of genetic disease (68.1%). Patients who declined a second genetic counseling session were more likely to terminate than those who had the additional session (70.2% vs. 55.7%). Indications for procedure, type of ultrasound findings, and number of living children were evenly distributed throughout this study population.

**TABLE 2:** Characteristics of the study population.

Characteristic	Total *n* (%_col_) (or mean ± SD)	Continued pregnancy *n* (%_col_) (or mean ± SD)	Terminated pregnancy		*p*‐Value
			*n* (%_col_) (or mean ± SD)	%_row_	
All patients	332 (100.0)	128 (100.0)	204 (100.0)	61.5	
Maternal age^a^					
<35	164 (49.4)	75 (58.6)	89 (43.6)	54.3	0.008
≥35	168 (50.6)	53 (41.4)	115 (56.4)	68.5	
Paternal age^b^					
<40	240 (72.3)	94 (73.5)	146 (71.6)	60.8	0.282
≥40	86 (25.9)	30 (23.4)	56 (27.4)	65.1	
Unknown	6 (1.8)	4 (3.1)	2 (1.0)	33.3	
Maternal race/ethnicity					
AAPI	55 (16.6)	17 (13.3)	38 (18.6)	69.1	0.004
Black	31 (9.3)	21 (16.4)	10 (4.9)	32.3	
Hispanic	81 (24.4)	36 (28.1)	45 (22.1)	55.6	
MENA	9 (2.7)	4 (3.1)	5 (2.4)	55.6	
Unknown	1 (0.3)	0 (0.0)	1 (0.5)	100.0	
White	155 (46.7)	50 (39.1)	105 (51.5)	67.7	
Paternal race/ethnicity					
AAPI	53 (16.0)	20 (15.6)	33 (16.2)	62.3	<0.001
Black	36 (10.8)	25 (19.5)	11 (5.4)	30.6	
Hispanic	68 (20.5)	32 (25.0)	36 (17.6)	52.9	
MENA	8 (2.4)	3 (2.4)	5 (2.4)	62.5	
Unknown	4 (1.2)	1 (0.8)	3 (1.5)	75.0	
White	163 (49.1)	47 (36.7)	116 (56.9)	71.2	
Diagnostic procedure					
Amniocentesis	144 (43.4)	83 (64.8)	61 (29.9)	42.4	<0.001
CVS	188 (56.6)	45 (35.2)	143 (70.1)	76.1	
Indication for procedure					
Abnormal antenatal screen	132 (39.8)	46 (35.9)	86 (42.2)	65.2	0.047
Ultrasound findings	127 (38.2)	47 (36.7)	80 (39.2)	63.0	
Parental carrier status	52 (15.7)	21 (16.4)	31 (15.2)	59.6	
Information seekers	21 (6.3)	14 (11.0)	7 (3.4)	33.3	
Type of ultrasound finding(s)					
Multiple	69 (20.8)	28 (21.9)	41 (20.1)	59.4	0.158
Single	33 (9.9)	13 (10.2)	20 (9.8)	60.6	
Marker	101 (30.4)	30 (23.4)	71 (34.8)	70.3	
None	129 (38.9)	57 (44.5)	72 (35.3)	55.8	
Gestational age at diagnosis (weeks)^c^	16.5 ± 4.5	19.1 ± 4.7	15.0 ± 3.5	–	<0.001
Previous termination(s)					
No	263 (79.2)	104 (81.2)	159 (77.9)	60.5	0.470
Yes	69 (20.8)	24 (18.8)	45 (22.1)	65.2	
History of 2+ SAB					
No	293 (88.3)	114 (89.1)	179 (87.7)	61.1	0.717
Yes	39 (11.7)	14 (10.9)	25 (12.3)	64.1	
Family history of genetic disease					
No	231 (69.6)	92 (71.9)	139 (68.1)	60.2	0.471
Yes	101 (30.4)	36 (28.1)	65 (31.9)	64.4	
Number of living children					
One	135 (40.7)	42 (32.8)	93 (45.6)	68.9	0.058
Multiple	85 (25.6)	35 (27.3)	50 (24.5)	58.8	
None	112 (33.7)	51 (39.9)	61 (29.9)	54.5	
Genetic counselor results session					
No	131 (39.5)	39 (30.5)	92 (45.1)	70.2	0.008
Yes	201 (60.5)	89 (69.5)	112 (54.9)	55.7	

Abbreviations: AAPI, Asian, Asian American, and Pacific Islander; CVS, chorionic villus sampling; MENA, Middle Eastern and North African; SAB, spontaneous abortion.

^a^ The maternal ages ranged from 16 to 46 years old.

^b^ The paternal ages ranged from 20 to 51 years old.

^c^ Gestational age at diagnosis ranged from 11 to 35 weeks: 11–26 weeks for those who terminated the pregnancy and 11–35 weeks for those who continued the pregnancy.

When evaluating each of the characteristics independently, there were significant differences in termination rates for maternal age (*p* = 0.008), maternal race and ethnicity (*p* = 0.004), paternal race and ethnicity (*p* < 0.001), diagnostic procedure (*p* < 0.001), indication for procedure (*p* = 0.047), gestational age at diagnosis (*p* < 0.001), and whether there was a results session with a genetic counselor (*p* = 0.008). However, the multivariable logistic regression provides an understanding of the impact of these variables together, which better represents the multi‐faceted decision‐making process.

The number of pregnancy terminations in each prenatal diagnostic category was recorded (Table 3). The highest termination rates were observed for diagnoses of trisomy 18/trisomy 13/triploidy (81.0%), trisomy 21 (79.6%), and unbalanced rearrangements/rare trisomies (79.0%). The lowest termination rates were observed for diagnoses of balanced rearrangements (0.0%) and CNVs with likely benign phenotypes (11.8%). Single‐gene disorders of severe/profound severity had a termination rate of 63.9%, and single‐gene disorders of moderate/mild severity had a termination rate of 33.3%. Cytomegalovirus (CMV) was the only teratogen reported in this study at a termination rate of 25.0% as this was the only teratogen identified in this cohort. The distribution of each diagnosis and corresponding termination rates per diagnostic procedure were also reported (Table A2). For the majority of the diagnoses, a more significant proportion of patients terminated after a CVS as compared to amniocentesis (*p* < 0.001), including trisomy 21 (56.5% vs. 23.2%), trisomy 18/trisomy 13/triploidy (62.1% vs. 19.0%), and sex chromosome abnormalities (44.0% vs. 4.0%).

**TABLE 3:** Termination rates by prenatal diagnostic category.

Prenatal diagnosis	Total *n*	Terminated *n* (%_row_)
Trisomy 21	108	86 (79.6)
Trisomy 18/trisomy 13/triploidy	58	47 (81.0)
Sex chromosome abnormalities	25	12 (48.0)
Unbalanced rearrangements/rare trisomies	19	15 (79.0)
Balanced rearrangements^a^	7	0 (0.0)
Profound/severe single‐gene disorders	36	23 (63.9)
Moderate/mild single‐gene disorders	9	3 (33.3)
CNVs with known clinical phenotype	18	8 (44.4)
CNVs with variable phenotype	15	4 (26.7)
CNVs with likely benign phenotype	17	2 (11.8)
Regions of homozygosity	16	3 (18.8)
Cytomegalovirus	4	1 (25.0)
All patients	332	204 (61.5)

Abbreviation: CNV, copy number variant.

^a^ The balanced rearrangements consist of two de novo rearrangements and five familial rearrangements.

In a bivariate analysis, the association between elective termination and the type of fetal diagnosis compared with sex chromosome abnormalities was assessed without accounting for other variables (Table A3). Sex chromosome abnormalities were used as the comparison to mirror prior studies that evaluated pregnancy termination rates (Hawkins et al., 2013). The highest odds of termination of pregnancy when compared with sex chromosome abnormalities were trisomy 18/trisomy 13/triploidy (Odds ratio [OR] 4.27, 95% Confidence interval [CI] 2.22–8.24), trisomy 21 (OR 3.91, 95% CI 2.45–6.24), and unbalanced rearrangements/rare trisomies (OR 3.75, 95% CI 1.24–11.30). Prenatal diagnosis of CNVs with likely benign phenotype (OR 0.17, 95% CI 0.03–0.99) and regions of homozygosity (OR 0.23, 95% CI 0.07–0.81) had the lowest odds of terminating the pregnancy.

After backward elimination, the final multivariable logistic regression model included the type of fetal diagnosis, paternal race and ethnicity, and gestational age at diagnosis (Table 3). The area under the receiver‐operating characteristic (AUC) of this regression was 0.86. Three of the prenatal diagnostic categories were significantly associated with elective termination. Compared with sex chromosome abnormalities, trisomy 21 (adjusted odds ratio [aOR] 4.39, 95% CI 1.58–12.24) and trisomy 18/trisomy 13/triploidy (aOR 6.35, 95% CI 1.93–20.90) had significantly higher odds to terminate, while CNVs with likely benign phenotype (aOR 0.17, 95% CI 0.03–0.99) had significantly lower odds to terminate.

Higher termination rates were reported for unbalanced rearrangements/rare trisomies (aOR 4.37, 95% CI 0.95–20.04), profound/severe single‐gene disorders (aOR 2.81, 95% CI 0.84–9.43), and CNVs with known clinical phenotype (aOR 1.11, 95% CI 0.28–4.38), these were not statistically significant. Lower termination rates were reported for CNVs with variable phenotype (aOR 0.29, 95% CI 0.06–1.31), regions of homozygosity (aOR 0.52, 95% CI 0.10–2.77), moderate/mild single‐gene disorders (aOR 0.53, 95% CI 0.09–3.11), and cytomegalovirus (aOR 0.74, 95% CI 0.06–9.45), these were also not statistically significant.

**FIGURE A1:**
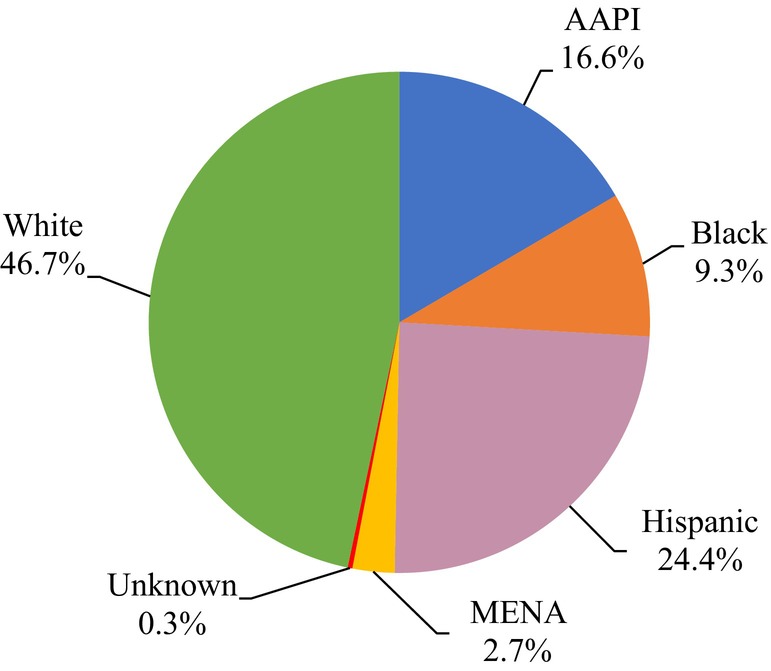
Racial and ethnic diversity of maternal study population. AAPI [Asian, Asian American, and Pacific Islander] (*n* = 55), Black (*n* = 31), Hispanic (*n* = 81), MENA [Middle Eastern and North African] (*n* = 9), Unknown (*n* = 1), and White (*n* = 155).

**FIGURE A2:**
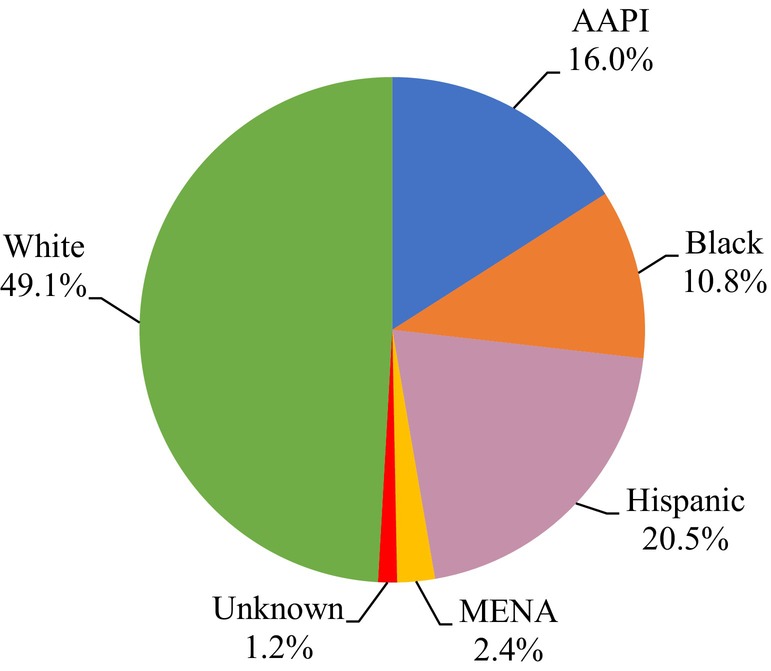
Racial and ethnic diversity of paternal study population. AAPI [Asian, Asian American, and Pacific Islander] (*n* = 53), Black (*n* = 53), Hispanic (*n* = 68), MENA [Middle Eastern and North African] (*n* = 8), Unknown (*n* = 4), and White (*n* = 163).

The maternal and paternal race/ethnicities are diverse in this patient population (Figures A1 and A2). Of those that terminated, the majority were of White race and ethnicity (maternal 51.5%, paternal 56.9%), followed by Hispanic (maternal 22.1%, paternal 17.7%), AAPI (maternal 18.6%, paternal 16.2%), Black (maternal 4.9%, paternal 5.4%), MENA (maternal 2.5%, paternal 2.5%), and Unknown (maternal 0.5%, paternal 1.5%). The lowest termination rates by race and ethnicity for both maternal and paternal groups were Black race and ethnicity (maternal 32.3%, paternal 30.6%). When controlling for other variables through multivariable logistic regression (Table 4), paternal race and ethnicity remained a predictor of the outcome. Paternal Black individuals were the least likely to terminate a pregnancy compared with their White counterparts (aOR 0.08, 95% CI 0.03–0.23).

**TABLE 4:** Multivariable logistic regression of factors evaluated for association with termination^a^.

Factor	Adjusted odds ratio	95% confidence interval
Prenatal diagnosis of sex chromosome abnormality compared with		
Trisomy 21	4.39	1.58–12.24
Trisomy 18/trisomy 13/triploidy	6.35	1.93–20.90
Unbalanced rearrangements/rare trisomies	4.37	0.95–20.04
Balanced rearrangements^b^	–	
Profound/severe single‐gene disorders	2.81	0.84–9.43
Moderate/mild single‐gene disorders	0.53	0.09–3.11
CNVs with known clinical phenotype	1.11	0.28–4.38
CNVs with variable phenotype	0.29	0.06–1.31
CNVs with likely benign phenotype	0.17	0.03–0.99
Regions of homozygosity	0.52	0.10–2.77
Cytomegalovirus	0.74	0.06–9.45
Paternal race/ethnicity of white compared with		
AAPI	1.24	0.51–3.00
Black	0.12	0.04–0.31
Hispanic	0.54	0.25–1.15
MENA	1.00	0.14–7.09
Unknown	0.73	0.07–7.87
Gestational age at diagnosis (weeks)	0.84	0.78–0.90

Abbreviations: AAPI, Asian, Asian American, and Pacific Islander; CNV, copy number variant; MENA, Middle Eastern and North African.

^a^ The area under the receiver‐operating characteristic (AUC) of this regression was 0.86.

^b^ All pregnancies with a balanced rearrangement did not terminate, so this variable could not be assessed in logistic regression.

Overall, the average gestational age at diagnosis was 16.5 ± 4.5 weeks. For those who terminated, the average gestational age at diagnosis was less than that of those who continued the pregnancy (15.0 ± 3.5 weeks vs. 19.1 ± 4.7 weeks). When controlling for other variables (Table 4), gestational age at diagnosis predicted elective termination (aOR 0.84, 95% CI 0.78–0.90). For each 1‐week increase in gestational age at diagnosis, patients were 1.2 times less likely to terminate the pregnancy.

## 4 DISCUSSION

Overall, 61.5% of the study population terminated a pregnancy with a fetal diagnosis. This overall termination rate is lower than expected given rates reported previously in the literature. Prior studies that only reported on diagnoses of trisomies and sex chromosome abnormalities reported overall termination rates around 81.0%–82.9% (Hawkins et al., 2013; Shaffer et al., 2006). The inclusion of CNVs and single‐gene disorders lowers the overall termination rate identified in this cohort.

Three factors were identified as predictors of the decision to terminate an affected pregnancy: type of diagnosis, paternal race and ethnicity, and gestational age at diagnosis. The termination rates reported per fetal diagnosis are consistent with previous reports (Table 5). A termination rate of 79.6% for Trisomy 21 diagnoses was to be expected in this study, given previous reports of 80.0–93.5% termination rates for Trisomy 21 in prior studies (Balkan et al., 2010; Hawkins et al., 2013; Hume & Chasen, 2015; Shaffer et al., 2006; Suzumori et al., 2015). Similar to prior studies, lower termination rates were observed for sex chromosome abnormalities compared to trisomy 21 and other common trisomies (Balkan et al., 2010; Hawkins et al., 2013; Shaffer et al., 2006; Suzumori et al., 2015). Sex chromosome aneuploidies are considered to be less severe phenotypically than common trisomies, which may explain the difference in termination rates. Stern et al. (2021) reported higher termination rates for pathogenic/likely pathogenic CNVs when compared with variants of uncertain significance CNVs (55.6% vs. 17.8%), which is similar to the difference in termination rates for CNVs in our study. These observed differences in termination rates per diagnosis may be attributed to differences in phenotypic severity, where more severe phenotypes have higher termination rates (Hawkins et al., 2013; Stern et al., 2021).

**TABLE 5:** Termination rates per prenatal diagnosis compared with previous studies.

Prenatal diagnosis	Current study termination rate (%)	Previous studies	
		Termination rate (%)	Study and location
Trisomy 21	79.6	93.5	Suzumori et al. (2015), Japan
		87.0	Hawkins et al. (2013), USA
		87.0	Shaffer et al. (2006), USA
		81.0	Hume and Chasen (2015), USA
		80.0	Balkan et al. (2010), Turkey
Trisomy 18/trisomy 13^a^	78.0	100.0	Balkan et al. (2010), Turkey
		100.0	Suzumori et al. (2015), Japan
		94.0	Hawkins et al. (2013), USA
		84.0	Shaffer et al. (2006), USA
		73.0	Hume and Chasen (2015), USA
Sex chromosome abnormality	48.0	60.0	Balkan et al. (2010), Turkey
		60.0	Shaffer et al. (2006), USA
		46.7	Suzumori et al. (2015), Japan
		43.0	Hawkins et al. (2013), USA
CNVs with known clinical phenotype	44.4	55.6	Stern et al. (2021), Israel
CNVs with variable phenotype	26.7	58.0	Shi et al. (2023), China
		17.8	Stern et al. (2021), Israel
Single‐gene disorders^b^	57.8	71.0	Bruwer et al. (2022), Oman

Abbreviation: CNV, copy number variant.

^a^ Termination rate adjusted to reflect only trisomy 18 and trisomy 13, excluding triploidy for comparison to other studies.

^b^ Termination rate adjusted to reflect the combined rate of all single‐gene disorders for both profound/severe and moderate/mild single‐gene disorder categories.

Differences in termination rates by phenotypic severity are also demonstrated within the rates for single‐gene disorders. A variety of single‐gene disorders were identified in this cohort (Table A1). Severe/profound single‐gene disorders identified in this cohort included Thanatophoric Dysplasia, Spinal Muscular Atrophy, and Fragile X Syndrome. Moderate/mild single‐gene disorders identified included Hemophilia A, Usher Syndrome Type 2, and homozygous GJB2. Single‐gene disorders of severe/profound severity had higher termination rates than those of moderate/mild severity (63.9% vs. 33.3%). Given that termination rates of single‐gene disorders are just beginning to be reported, it is important to assess these rates in terms of fetal diagnoses that have been reported for many years. In this study, severe/profound single‐gene disorders reported almost twice the termination rate of moderate/mild single‐gene disorders. Similarly, in this study, trisomy 21 and trisomy 18/trisomy 13/triploidy reported almost double the termination rate of sex chromosome abnormalities (79.6% and 81.0% vs. 48.0%). Compared with another study, the termination rate for all single‐gene disorders in our study was lower than reported by Bruwer et al. (2022) (57.8% vs. 71.0%). The difference may be attributed to differing patient populations. However, more research on termination rates of single‐gene disorders is needed to better understand these rates.

Fetal diagnoses that were predicted to have no impact on phenotype, including balanced rearrangements and CNVs with likely benign phenotypes, had the lowest reported termination rates (0.0% and 11.76%). As expected, the two balanced rearrangements that were de novo had no ultrasound anomalies that indicated prenatal diagnostic testing. Their indications were abnormal carrier screening and information‐seekers. It was unexpected, however, that there were reported terminations for diagnoses of CNVs with likely benign phenotypes. The two individuals that terminated a pregnancy with a likely benign CNVs did have ultrasound anomalies reported which were also the indication for the prenatal diagnostic testing. While the type of ultrasound finding was a characteristic evaluated in this study and not found to be statistically significant for the entire cohort, it may have a larger impact in the setting of a benign diagnostic finding.

Within paternal race and ethnicity, Black and Hispanic individuals had the lowest termination rates when compared with White individuals in multivariable logistic regression. Similar trends have been observed for maternal race and ethnicity. However, paternal race and ethnicity are rarely assessed in the context of pregnancy termination. Previous publications have demonstrated the impact of paternal race and ethnicity on other prenatal outcomes (Palatnik et al., 2021) and levels of medical mistrust (Armstrong et al., 2007). Race and ethnicity may have acted as a proxy for other unassessed variables, like socio‐economic status, income level, education, and current health status, which influence health outcomes (Williams et al., 2016). Race and ethnicity may no longer be associated if additional socioeconomic factors had been collected.

The average gestational age at diagnosis was earlier for those who terminated compared with those who continued, which has been observed in other studies (Hawkins et al., 2013; Rauch et al., 2005). Gestational age at diagnosis may be a predictor of the decision to terminate because, as gestational age increases, pregnant people report feeling a greater sense of attachment to the pregnancy (Lumley, 1982). Similarly, it may be a more difficult decision to terminate after feeling movement during the pregnancy, which can begin as early as 16 weeks gestation (American Pregnancy Association, n.d.), while the average gestational age at diagnosis for those who terminated in this study was 15 weeks. This underscores the importance of early diagnosis, especially after the implications of the Dobbs decision (Haberman et al., 2024). While the type of diagnostic procedure was not a significant predictor in the multivariable logistic regression, it was observed that a more significant proportion of patients terminated after a CVS as compared to amniocentesis. Given that CVS occurs at earlier gestational ages than amniocentesis, this trend could be reflective of the significant relationship reported of higher termination rates for earlier gestational ages at diagnosis.

A few factors identified as predictors of elective termination in prior studies did not remain in our final model, including prior termination history (Hawkins et al., 2013) and multiple ultrasound anomalies (Rauch et al., 2005). In this study, it is possible that pregnancies with multiple anomalies terminated before results disclosure; therefore, excluding those patients from this study. Higher termination rates have been reported for individuals of advanced maternal age (≥35 years), as well as after a CVS compared with amniocentesis (Shaffer et al., 2006). Maternal age is a variable that, while significant in prior studies, was speculated to be a proxy for multiple variables that may become more common with age, including number of current children and history of previous terminations that were also assessed in this study, with no significant differences identified. Hawkins et al. (2013) also reported that those declining an additional genetic counseling session were more likely to terminate, likely because those individuals were definitive about their decision after the disclosure of the initial result.

Methodological limitations are noted. Despite this being one of the largest studies to look at factors associated with termination based on genetic diagnosis, the overall frequency of rare diagnoses may have inhibited the regression analysis from identifying additional predictors in the final model. Another limitation is that this study does not reflect those who may identify with multiple races and ethnicities. Education (Ba et al., 2023), socioeconomic status (Jones et al., 2002), and religion (Balkan et al., 2010) have been reported as significant factors in the decision to terminate an affected pregnancy. However, these factors could not be assessed in this study.

A strength of this study is its population diversity and generalizability of results due to the racial and ethnic distribution that is reflective of New Jersey, according to the 2020 United States Census Bureau (2020). Other strengths of this study include the overall sample size, the quality and consistency of chart notes at a single institution that contributed to little missing data, and the various characteristics assessed.

The findings from this study are important and impactful for the practice of genetic counseling. To provide the best patient care, a genetic counselor needs to be able to understand any of the factors that are influencing an individual's decision‐making. Understanding what factors may influence pregnancy termination decisions following the disclosure of a genetic diagnosis helps genetic counselors provide the best education and assistance towards a patient's informed decision regarding their pregnancy management. This information can help a genetic counselor identify when their patient has clinical and/or demographic factors that significantly impact the decision to terminate an affected pregnancy, which may allow the counselor to have improved preparation and anticipation of the questions and needs of their patient.

Existing literature on termination rates has focused primarily on common aneuploidies. Minimal research has been conducted on other prenatal diagnoses with decisions regarding pregnancy termination. While a limitation of this study is that sample sizes for other fetal diagnoses were small, this study adds to the existing literature and helps to build the foundation for future research that may focus on termination rates of types of fetal diagnoses that have been less studied, including copy number variants and single‐gene disorders. Collaboration with multiple centers could allow for a larger sample size for these types of diagnoses. More reports of these diagnoses and termination rates will aid in the interpretation of those reported in this study, as well as in understanding the decision‐making of those who receive these diagnoses. The inclusion of other factors, like education, socioeconomic status, and religion, is important to assess other potential influences on the decision to terminate an affected pregnancy. In future research, socioeconomic status could be assessed indirectly through type of insurance, which would provide more insight into the financial impact on decision‐making.

In conclusion, we reported termination rates for a variety of fetal diagnoses, including CNVs and single‐gene disorders. Three factors were identified as predictors of the decision to terminate an affected pregnancy: type of diagnosis, paternal race and ethnicity, and gestational age at diagnosis. Terminating an affected pregnancy can be a difficult decision. This research can help providers understand what influences their patients and what factors impact their decision making.

## AUTHOR CONTRIBUTIONS

All listed authors substantially contributed to the manuscript and agreed to the final submitted version. The concept of the article was proposed by Elena Ashkinadze, and the design of this study was created by all listed authors in collaboration. Data collection was completed by Madeline J. Herman. The data analysis was completed by Madeline J. Herman, Gary A. Heiman, and Emily B. Rosenfeld. The initial draft of the manuscript was prepared by Madeline J. Herman, with the interpretation of the data and editing of the manuscript completed by all listed authors.

## FUNDING INFORMATION

No funding was acquired for this research.

## CONFLICT OF INTEREST STATEMENT

The authors have no conflicts of interest and nothing to disclose.

## ETHICS STATEMENT

Human studies and informed consent: This research was approved by the Rutgers University Institutional Review Board (Study ID: Pro2023000800) on June 6, 2023.

Animal studies: No non‐human animal studies were conducted.

## PATIENT CONSENT STATEMENT

A waiver of patient consent was approved by the Rutgers University Institutional Review Board (Study ID: Pro2023000800) on June 6, 2023. This is a retrospective study with minimal risks to the study subjects, and all subjects are over age 18.

## Data Availability

The data supporting this study's findings are available on request from the corresponding author. The data are not publicly available due to privacy or ethical restrictions.

## APPENDIX A

**TABLE A1:** Breakdown of prenatal diagnoses per category.

Prenatal diagnosis	Total *n*	Terminated pregnancy *n* (%)
Trisomy 21	**108**	**86 (79.63)**
Trisomy 21	105	84
Mosaic Trisomy 21	2	1
Trisomy 21 with Xp22.31 dup	1	1
Trisomy 18/trisomy 13/triploid	**58**	**47 (81.03)**
Trisomy 18	27	25
Trisomy 13	20	13
Triploid	7	7
Mosaic trisomy 18	1	0
Mosaic trisomy 13	1	1
Mosaic triploid	1	1
48,XXY+18	1	0
Sex chromosome abnormalities	**25**	**12 (48.00)**
45X	11	8
Mosaic 45X	2	0
46XY/46XX	1	0
47XXX	4	2
47XXY	3	1
47XXY and 16p11.2 dup	1	0
47XYY	2	0
48XXYY	1	1
Unbalanced rearrangements/rare trisomies	**19**	**15 (78.95)**
46,XY,t(14;21)(q10;q10)+21	1	1
46,XY,t(9;15)(q21.3;q13)−15	1	1
46,XX,+21,der(21;21)(q10;q10)/45,XX,−21	1	1
46, XY,der(3)t(3;4)(p26.2;15.2)	1	1
46,XX,der(13)t(13;15)(q21.32;q13),−15	1	1
46,XX,der(15)t(15;8)	1	1
46,XX,der(3)t (3, 21)(q29;q11.2)	1	1
46,XX,der(3;5)(q26.2;p15.1)	1	1
46,XY,der(14;21)+21	1	1
46,XY,der(5)t(3;5)	1	1
46,XY,der(5)t(3;5)(q26.2;p15.1)	1	1
46,XY,der(9)t(9;13)(p23;q34)	1	1
46,XY,inv. (9)(p11q13),der(12)ins(12;4)(q24.1;q27q33)	1	1
46,X,inv.(X)(p22.3p11.3)	1	1
Trisomy 16	1	0
Trisomy 22	1	1
Trisomy 8 and trisomy 9	1	0
Mosaic trisomy 8	1	0
Mosaic trisomy 10 and trisomy 20	1	0
Balanced rearrangements	**7**	**0 (0.00)**
45,XY,t(13;14)(q10;q10)	1	0
46,XX,t(2;11)(q32;q23)	1	0
46,XX,t(4;5)(q31.3;p15.3)	1	0
46,XX,t(8;22)(q24.1;q11.2)	1	0
46,XX,inv. (9)(p12q13)	1	0
46,XY,inv. (1)(p32q42)	1	0
46,XY,inv. (2)(p11.2;q13)	1	0
Profound/severe single‐gene disorders	**36**	**23 (63.89)**
Profound		
Thanatophoric dysplasia	2	1
Severe		
Alpha thalassemia	1	0
Bartsocas‐Papas	1	0
Beckwith‐Wiedemann syndrome	1	0
Beta thalassemia	2	2
Campomelic dysplasia	1	1
Cystic fibrosis	4	3
Duchenne muscular dystrophy	1	0
Familial dysautonomia	1	1
Fragile X	3	3
Huntington's disease	7	7
Myotonic dystrophy	2	1
Noonan syndrome	3	0
Osteogenesis imperfecta	1	0
Renpenning syndrome	1	1
Sickle cell disease	3	1
Spinal muscular atrophy	2	2
Moderate/mild single‐gene disorders	**9**	**3 (33.33)**
Moderate		
GJB2 homozygous	1	0
Neurofibromatosis type 1	2	1
Neurofibromatosis type 2	1	1
NOTCH1	1	0
RECQL4 homozygous	1	1
Usher syndrome type 2	1	0
Mild		
Hemophilia A	1	0
Pseudocholinesterase deficiency	1	0
CNVs with known clinical phenotype	**18**	**8 (44.44)**
2q37 del	1	1
4p del	1	1
5p14 del	1	1
5q11.2‐12.3 del	1	1
6pter‐p25.3 del AND 6p25.3‐p22.2 dup	1	1
7q31.1‐q36 del	1	0
8p23.3 del	1	0
10q dup	1	0
11p15.5 dup	1	0
14q32.1 del	1	0
16p13.11 del	1	0
17p12 dup	1	0
17q12 del interstitial	1	0
18q21.1 del	1	1
18q22.1‐18q23 del	1	1
22q11.2 dup	1	0
22q12 del	1	1
SRY exon 1 del	1	0
CNVs with variable phenotype	**15**	**4 (26.67)**
1q21.3 dup interstitial	1	0
2p del	1	1
3q29 dup	1	0
6q26 del interstitial	1	0
7p15.2‐14.1 del interstitial	1	1
9q34.3 dup	1	1
12p12.1 del interstitial	1	0
15q dup	1	0
15q13.1‐13.3 dup interstitial	1	0
16q23 del	1	0
17p13.2 del interstitial	1	0
20q13.3 dup interstitial	1	0
21q21 del	1	1
22q11.22–23 del	1	0
Xp21 dup	1	0
CNVs with likely benign phenotype	**17**	**2 (11.76)**
1q21.1‐q21.1 del	1	0
2q13 del	3	0
2q13‐q13 del interstitial	1	1
3q29 dup	1	0
5q13.3 del	1	0
11p15.4 del	1	0
13q31.1 del	1	0
15q11.2‐13.1 dup interstitial	1	0
16p12 del	1	0
16p12.2 del interstitial	1	0
16p12.2 dup interstitial	1	0
16p13.11 dup	1	1
16q24.3 dup	1	0
Xp21.1 dup	1	0
Regions of homozygosity	**16**	**3 (18.75)**
Regions of homozygosity	14	3
Uniparental disomy	2	0
Cytomegalovirus	**4**	**1 (25.00)**
Cytomegalovirus	4	1
Total	332	204 (61.45)

*Note:* Bold values indicate categorical totals.

Abbreviation: CNV, copy number variant.

**TABLE A2:** Termination rates per prenatal diagnostic category and type of procedure.

Prenatal diagnosis	Total *n*	CVS		Amniocentesis	
		Total *n* (%_row_)	Terminated *n* (%_row_)	Total *n* (%_row_)	Terminated *n* (%_row_)
Trisomy 21	108	67 (62.0)	61 (56.5)	41 (38.0)	25 (23.2)
Trisomy 18/trisomy 13/triploidy	58	41 (70.7)	36 (62.1)	17 (29.3)	11 (19.0)
Sex chromosome abnormalities	25	16 (64.0)	11 (44.0)	9 (36.0)	1 (4.0)
Unbalanced rearrangements/rare trisomies	19	15 (79.0)	11 (57.9)	4 (21.1)	4 (21.1)
Balanced rearrangements^a^	7	4 (57.1)	0 (0.0)	3 (42.9)	0 (0.0)
Profound/severe single‐gene disorders	36	18 (50.0)	15 (41.7)	18 (50.0)	8 (22.2)
Moderate/mild single‐gene disorders	9	4 (44.4)	3 (33.3)	5 (55.6)	0 (0.0)
CNVs with known clinical phenotype	18	4 (22.2)	2 (11.1)	14 (77.8)	6 (33.3)
CNVs with variable phenotype	15	10 (66.7)	4 (26.7)	5 (33.3)	0 (0.0)
CNVs with likely benign phenotype	17	7 (41.2)	0 (0.0)	10 (58.8)	2 (11.8)
Regions of homozygosity	16	2 (12.5)	0 (0.0)	14 (87.5)	3 (18.8)
Cytomegalovirus	4	0 (0.0)	0 (0.0)	4 (100.0)	1 (25.0)
All patients	332	188 (56.6)	143 (43.1)	144 (43.4)	61 (18.4)

Abbreviations: CNV, copy number variant; CVS, chorionic villus sampling.

^a^ The balanced rearrangements consist of two de novo rearrangements and five familial rearrangements.

**TABLE A3:** Bivariate logistic regression of prenatal diagnostic categories for association with termination.

Prenatal diagnosis	Odds ratio	95% confidence interval
Sex chromosome abnormality compared with		
Trisomy 21	4.39	1.58–12.24
Trisomy 18/trisomy 13/triploidy	6.35	1.93–20.90
Unbalanced rearrangements/rare trisomies	4.37	0.95–20.04
Balanced rearrangements^a^	–	
Profound/severe single‐gene disorders	2.81	0.84–9.43
Moderate/mild single‐gene disorders	0.53	0.09–3.11
CNVs with known clinical phenotype	1.11	0.28–4.38
CNVs with variable phenotype	0.29	0.06–1.31
CNVs with likely benign phenotype	0.17	0.03–0.99
Regions of homozygosity	0.52	0.10–2.77
Cytomegalovirus	0.74	0.06–9.45

Abbreviation: CNV, copy number variant.

^a^ All pregnancies with a balanced rearrangement did not terminate; so this variable could not be assessed in logistic regression.
